# Secondary-Transferring Graphene Electrode for Stable FOLED

**DOI:** 10.1186/s11671-018-2767-z

**Published:** 2018-11-06

**Authors:** Yunjie Teng, Shoufeng Tong, Min Zhang

**Affiliations:** 1 College of Opto-Electronic Engineering, Changchun University of Science and Technology, Changchun, 130012 People’s Republic of China; 2 Institute of Space Photo-Electronic Technology, Changchun University of Science and Technology, Changchun, 130012 People’s Republic of China

**Keywords:** Graphene, Sharp winkles, Secondary-transferring, Flexible organic light emitting device

## Abstract

In this work, sharp wrinkles on graphene films, caused by graphene duplicating the grain boundary cracks of copper foil during the preparation process, were carefully explored. A secondary-transferring graphene film process was proposed to re-transform the “Peak” morphology of graphene surface into “Valley” form. The process we have developed is highly effective and almost nondestructive to the graphene through testing the surface morphology and photo-electric properties before and after the secondary-transferring process. Flexible organic light-emitting device (FOLED) with PEDOT:PSS/SLG/NOA63 framework as a targeted application was fabricated to illustrate the value of our proposed method in fabricating stable devices, the maximum luminance can reach about 35000 cd/m^2^, and the maximum current efficiency was 16.19 cd/A. This method can also be applied to the roll-to-roll preparation of large area high-quality graphene.

**Electronic supplementary material:**

The online version of this article (10.1186/s11671-018-2767-z) contains supplementary material, which is available to authorized users.

## Background

Graphene, arranged by single-layer carbon atoms in the shape of a unique hexagonal honeycomb lattice structure, is a promising two-dimensional transparent conductive material for flexible organic light-emitting device (FOLED) due to its excellent conductivity, high transmittance, and flexibility [1–3]. Jong has fabricated 30-in. graphene films by layer-by-layer stacking and measured its sheet resistance at values as low as ∼ 30 Ω/sq. and transparency at ∼ 90%, which is superior to commercial indium tin oxide (ITO) electrodes [4]. Chiu reported a high-mobility boron-doped graphene acting as an effective anode of FOLED with a record-high external quantum efficiency ~ 24.6% [5].

Graphene can be prepared by micromechanical exfoliation [6], electrolytic exfoliation of graphite [7], epitaxial growth [8, 9], chemical vapor deposition (CVD), and graphite oxide reduction [10, 11]. So far, CVD on copper is the most effective method for preparing high-quality large-scale graphene films, which grows mainly by the surface adsorption and catalytic processes reported by Ruoff—the carbon source was adsorbed onto the surface of copper foil, under the catalysis of copper, the carbon bonds break, and carbon atoms reform into sp^2^ hybridized graphene [12]. Once the surface of copper is completely covered with a single layer of graphene, the catalytic effect of copper is lost, and no more layers of graphene can be grown, so graphene grown on the surface of copper is to be likely a self-limiting process to realize uniform single-layer graphene (SLG).

However, it is well known that there are various atomic scale defects, large number of wrinkles, and especially artificial cracks and impurity residues on the copper foil-based SLG films [13–17]. Previous studies have already primarily yielded insight into reducing all above defects density in the preparation and transfer process. Joshua determined that Cu substrate crystallography affects graphene nucleation and growth more than facet roughness by growing graphene on polycrystalline Cu with different crystal direction, coming to the conclusion that Cu (111) surface promoted few defects SLG [18]. Avouris studied the structural morphology and electronic properties in CVD graphene wrinkles by quantum transport calculations and AFM images; the maximum collapsed wrinkle height can reach about 6 nm, and the local interlayer tunneling effect across the collapsed region contributed a significant resistance to the overall device [19]. Generally, by adjusting the parameters of CVD process [20], pretreatment of copper foil [21], and surface modification [22, 23], the density of defects can be reduced to a certain extent. However, compared with these defects in the preparation process and the wrinkles caused by the transfer process, little attention has been paid to the sharp wrinkles caused by graphene duplicating the grain boundary cracks of copper foil. These grain boundary cracks, produced in Cu foil pre-annealing treatment process, are the result of polycrystalline copper recrystallizing at high temperature to form larger-size single crystal domains. Since graphene is grown on the surface of Cu foil, its topography will completely replicate the surface structure of Cu foil, including the cracks. After transferring, the cracks of graphene on Cu foil will become the sharp wrinkles on the target substrate, so sharp wrinkles are ubiquitous and inevitable on graphene film, no matter what type of transfer process used, such as mediator-assisted transfer [24], direct dry and wet transfer [25], and mass production roll-to-roll transfer [26]; these sharp wrinkles undoubtedly cause large surface roughness of graphene films, resulting in a poor performance of organic devices, especially FOLED [27].

**Fig. 1:**
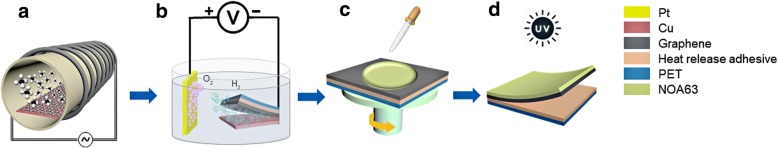
Design overview of synthesis and transfer processes for graphene film. **a** The CVD growth of graphene on Cu foil; CH_4_ was using as the carbon source. **b** Illustration of the first transfer process of bubbling off graphene from Cu substrate; PET coated with heat release adhesive (HRA) was used as support layer. The electrolyte was NaOH aqueous solution, Pt was used as anode, and PET/HRA/graphene/Cu foil was cathode. **c**, **d** Illustration of secondary-transferring graphene electrode. Drip and spin coat the UV-curable polymer NOA63 on graphene/HRA/PET substrate, then solidify NOA63 film and left it off from graphene/HRA/PET

In this paper, we used a fast and efficient bubble transfer method which can nondestructively transfer graphene from Pt or Cu substrate and no residual impurities while comparing with other transfer methods [28], then we explored graphene morphology after one-step transfer by optical microscope; the height of sharp wrinkles on graphene surface can reach hundreds of nanometers, which can easily lead the device deteriorate even breakdown. Therefore, we proposed a secondary-transferring graphene film process to re-transform the “Peak” morphology of graphene surface into “Valley” form using two organic components with different adhesion—PET coated with low adhesion heat release adhesive (HRA/PET) used as the first support layer, the adhesiveness of the HRA can decrease sharply to zero when temperature rises to about 100 °C, and NOA63 with high adhesion used as the second support layer; as shown in Fig. 1, the graphene film was transferred almost nondestructively to the flexible substrate. Finally, we illustrated the necessity of our proposed method in fabricating stable FOLED through contrasting experiments; this method can also be applied to the roll-to-roll preparation of large area high-quality graphene.

## Experimental Methods

Figure 1 shows the design overview of the synthesis and secondary-transferring processes of graphene film. Cu foil (25 μm thickness) was heated to 1040 °C to recrystallize for 30 min and then annealed for 30 min at 1040 °C with a 15-sccm H_2_ gas flow in the CVD chamber. CH_4_, used as the carbon source, was injected at a flow rate of 60 sccm for 30 min, then samples were fast cooled to room temperature, as shown in Fig. 1a. Figure 1b shows the first transfer process of bubbling off graphene from Cu substrate. 2mol/L NaOH aqueous solution was used as electrolyte; PET coated with heat release adhesive (HRA), purchased from Nitto Kogyo corporation, Japan, was pressed to graphene/Cu foil, used as the support layer, and connected with negative electrode. A Pt rod was connected with positive electrode, large number of H_2_ bubbles generated at the interface between the graphene and Cu foil and removed the graphene from the copper substrate. After electrolysis, graphene was transferred from copper foil to PET/HRA. Figure 1c, d illustrates the secondary-transferring progress. Firstly, UV-curable polymer NOA63 was dripped and spin coated onto the graphene/HRA/PET substrate; the speed was set at 300 rpm for 15 s, followed by 600 rpm for 15 s. Then, the sample was placed in UV environment (350–380 nm) for 4 min to solidify NOA63. During the UV curing, the adhesiveness of HRA disappear due to the increased ambient temperature. Therefore, NOA63 with strong adhesion can stick and support graphene film and graphene was almost nondestructively transferred to the NOA63.

## Results and Discussions

**Fig. 2:**
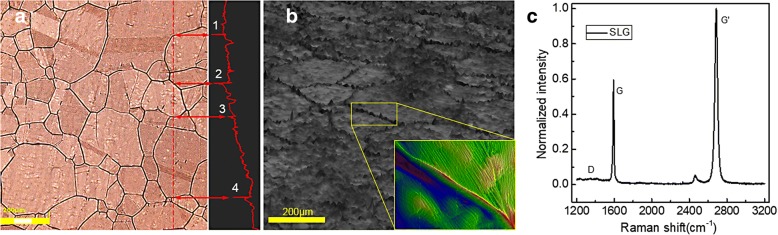
Three-dimensional laser confocal microscope map of **a** graphene on Cu foil and **b** graphene on HRA/PET. **c** Raman spectra of the graphene transferred from Cu foils onto SiO_2_/Si

To determine the quality of the graphene obtained, we performed optical microscope test and Raman measurements. Figure 2a shows the optical microscope map of graphene on Cu foil. The copper grains with size of 50–200 μm and the cracks were obviously observed after high-temperature annealing. It can be seen from the sectional view of the surface morphology that point 1–4 was grain boundary in crack form and they changed into sharp wrinkles after graphene bubbling-transferred onto HRA/PET; as shown in Fig. 2b, the inset map gave the three-dimensional morphology of the sharp wrinkles whose height can reach hundreds of nanometers. Figure 2c shows the Raman spectra of the graphene transferred from Cu foils onto SiO_2_/Si, a double-frequency Nd:YAG laser (532 nm) as excitation source. G band caused by the in-plane vibration of sp^2^ carbon atoms with a peak position ~ 1590 cm^−1^, and the G’ originated from two-phonon double resonance Raman process with a peak position ~ 2686 cm^−1^. Here, the intensity ratio of the G’ band to G band (*I*_G’_/*I*_G_) was 1.75 ± 0.015 (detailed data can be found in Additional file 1: Figure S1), which illustrated that the most of graphene we prepared was SLG [29]. Furthermore, the intensity ratio of D band to G band (*I*_D_/*I*_G_) quantified the structural defects and disorders of graphene; its value was just ~ 0.065, illustrating high quality of the prepared SLG [30].

**Fig. 3:**
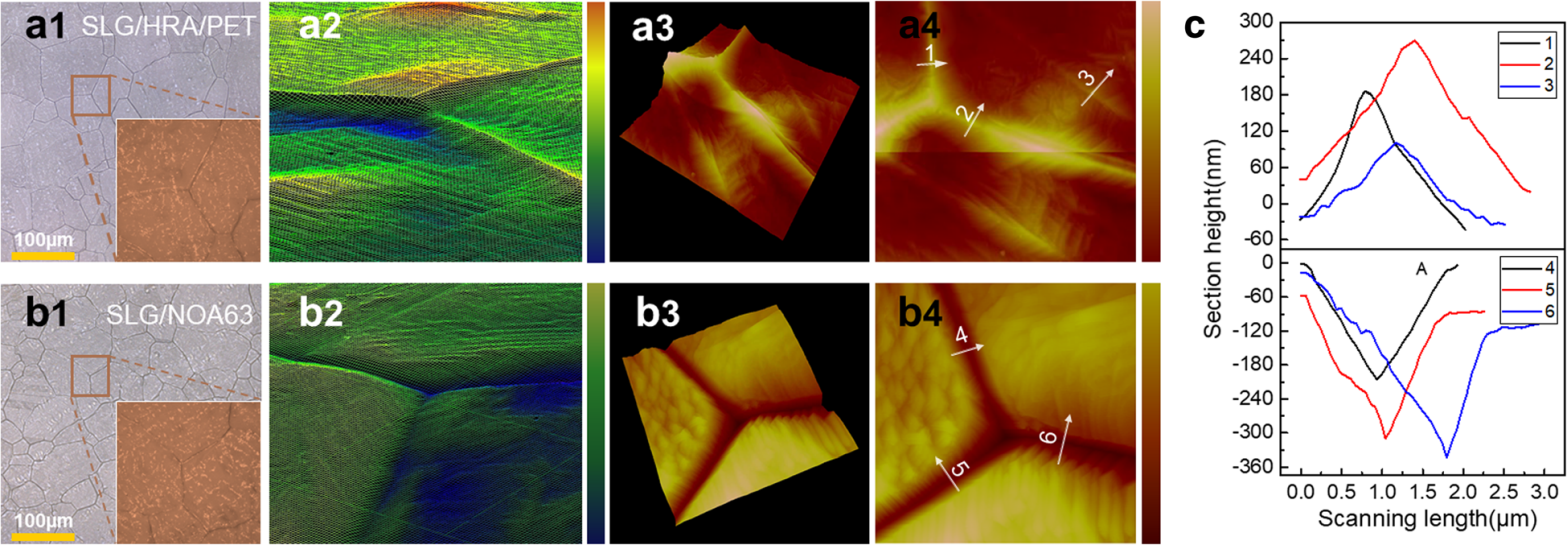
**a1** Two-dimensional plan maps of graphene on HRA/PET. **a2** Locally enlarged three-dimensional view of **a1**. **a3, a4** Three-dimensional AFM image and the corresponding two-dimensional map of graphene on HRA/PET. **b1** Two-dimensional plan maps of graphene on NOA63. **b2** Locally enlarged three-dimensional view of **b1**. **b3**, **b4** Three-dimensional AFM image and the corresponding two-dimensional map of graphene on NOA63. **c** Section height of AFM of point 1–6

**Fig. 4:**
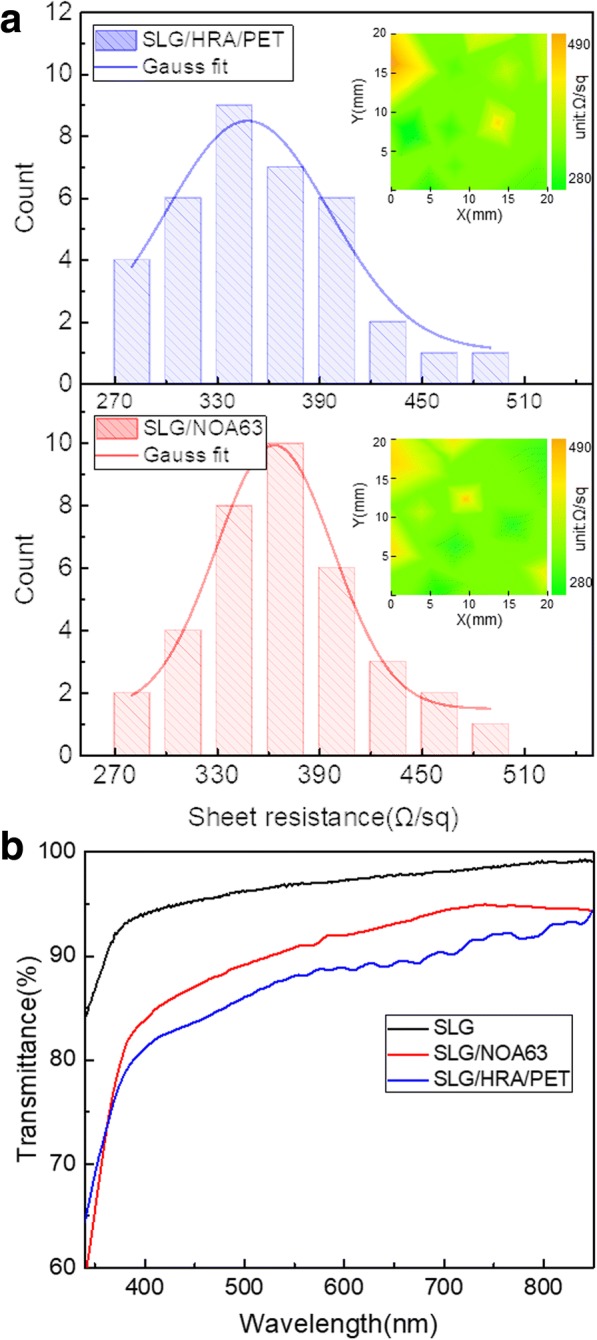
**a** Histogram and spatial distribution of sheet resistance of the SLG samples on HRA/PET and NOA63 (size 20 mm × 20 mm). **b** Transmittance of SLG, SLG/HRA/PET, and SLG/NOA63 in visible region; the thickness of HRA/PET and NOA63 are both about 150 μm

We further precisely explored the height of sharp wrinkles and the changes of surface morphology and photo-electric properties before and after the secondary-transferring. Figure 3a1–a4 shows the optical microscope and AFM measurement of SLG on HRA/PET; as previously mentioned, graphene replicated the surface morphology of copper foil, the grain boundary cracks became the sharp wrinkles as shown in locally enlarged Fig. 3a2. Section height of three-dimensional AFM image of point 1–3 shows that the height of sharp wrinkles on SLG can reach ~ 300 nm, which was harmful for stable FOLED. Figure 3b1–b4 shows the SLG film on NOA63; after the secondary-transferring, the sharp wrinkles on the graphene were returned into “Valley” form almost symmetrically and non-destructively, so the second transfer can be actually seen as a mirror reversal of graphene’s surface topography, as the point 1–3 showed in Fig. 3c. Figure 4a shows the maps and the histograms distribution of sheet resistance measured from 36 points of 20 mm × 20 mm of SLG on HRA/PET and NOA63; the sheet resistance of graphene film was measured by Van der Pauw technique, which was conducted by a four-point probe equipment connected to a source meter (Keithley 2400) under ambient conditions, the accuracy is 0.1 Ω/sq. As observed, the area corresponding to poor electrical qualities was attributed the non-close contact between HRA and graphene, where the graphene films were prone to holes or folds due to lack of supported substrate. However, there was almost no change in the distribution of sheet resistance before and after the secondary-transferring as the inset maps shown, and the average sheet resistance values of both were concentrated at about 360 Ω/sq. as seen by Gauss fit lines; this was mainly attributable to the strong adhesion of NOA63. Figure 4b shows the transmittance spectra of SLG, SLG/HRA/PET, and SLG/NOA63 in visible region; the thickness of HRA/PET and NOA63 were both about 150 μm for comparison purposes which were measured by thickness gauge (CHY-CA, Labthink International, Inc., China). The optical transmittance of them was respectively 96.6%, 88.1%, and 90.8% at 550 nm. It can be seen that NOA63 has higher transmittance than PET/HRA, which was beneficial to the light extraction of FOLED.

**Fig. 5:**
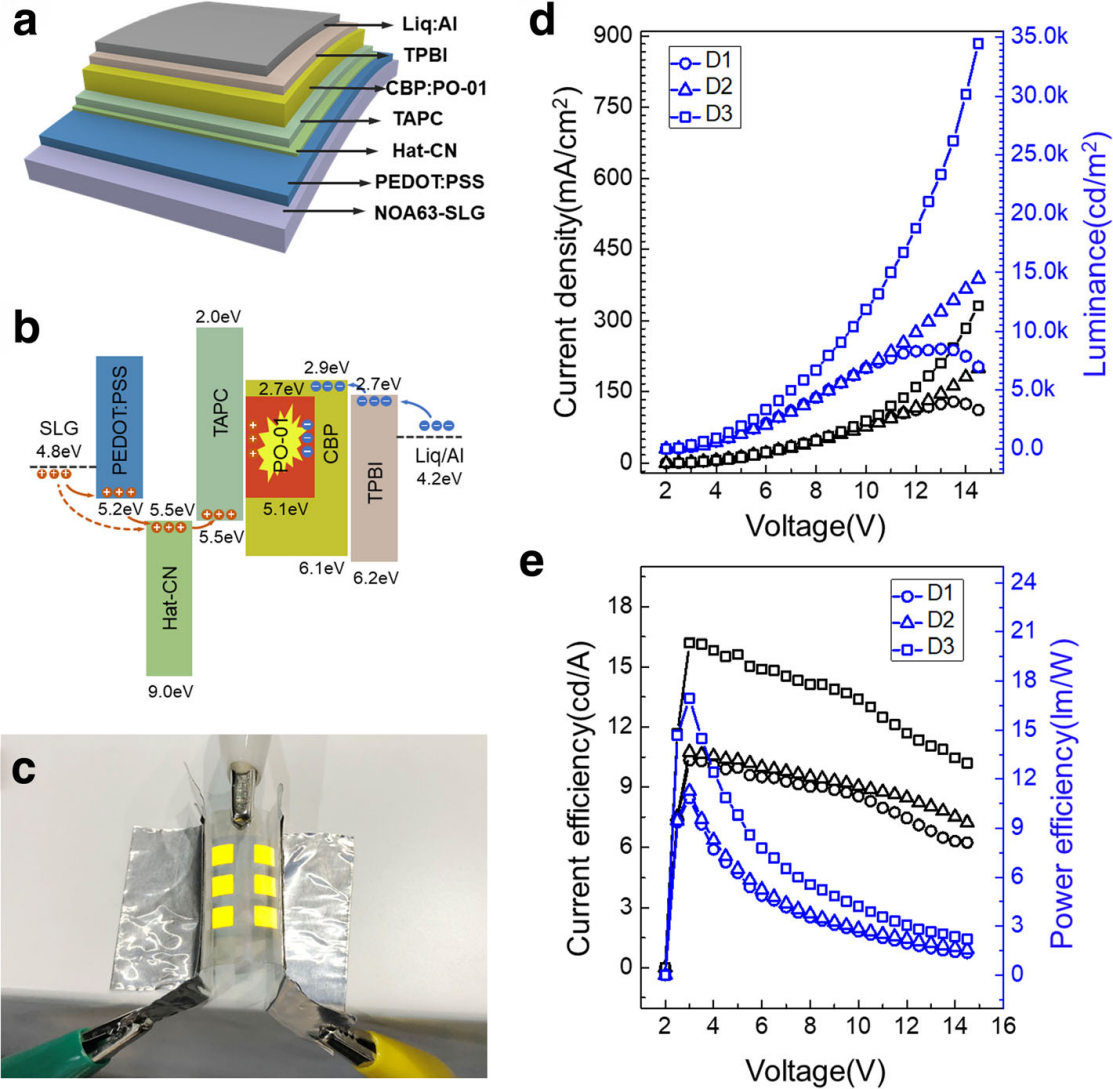
**a** Schematic device structure of the FOLED. **b** Work function of SLG and HOMO/LUMO energy level of the FOLED components. Device characteristics of D1(based on SLG/HRA/PET), D2(based on SLG/ NOA63), and D3(based on PEDOT:PSS/SLG/NOA63). **c** J-V-L characteristics. **d** Current efficiency and power efficiency-voltage characteristics. **e** Photograph of the FOLED based on SLG/NOA63 (size 4 mm × 4.5 mm × 6)

We fabricate the FOLEDs as a targeted application to explore the effectiveness of our secondary-transferring progress where the graphene acts as an anode. Figure 5a diagrammed the device structure of the FOLED, in which 10 nm Hat-CN was used as hole injection layer, 40 nm TAPC was the hole transport layer, 30 nm CBP doped with 10% PO-01 was light-emitting layer, 30 nm TPBI was electron transport layer, while 1 nm Liq and 100 nm Al were used as cathode. Considering the energy level matching at the graphene interface, we also introduced 50 nm PEDOT:PSS with the addition of 3 wt.% DMSO as the modified layer. On the one hand, PEDOT:PSS was liquid before the film is formed and smoothed the surface of SLG film by filling part of the “Valley.” On the other hand, it also reduced the barrier height between graphene and hole transport layer, as seen in Fig. 5b. The work function of SLG was 4.8 eV, measured by Kelvin probe system, the hole need surmount 0.7 eV to the lowest unoccupied molecular orbital (LUMO) of Hat-CN, while it only need to overcome 0.4 eV reaching the highest occupied molecular orbital (HOMO) of PEDOT:PSS; there was no doubt that it made the hole injection easier.

The optoelectronic characteristics, including current density-voltage-luminance (J-V-L) and current efficiency-voltage (CE-V) of the FOLEDs with/without secondary-transferring graphene electrode structure, are shown in Fig. 5c, d for device units D1 (based on SLG/HRA/PET), D2 (based on SLG/ NOA63), and D3 (based on PEDOT:PSS/SLG/NOA63). As we can see, D1 in which the graphene transferred by the first bubbling progress showed a significant drop in brightness and current density at a voltage of 13 V; as mentioned before, the sharp wrinkles existing on the surface of graphene caused the local current to short-circuit, making the FOLED unable to withstand a large current density. While D2 shows a stable upward trend, even if the voltage was high to 14.5 V with a luminance of ~ 15000 cd/m^2^, this was attributed to the decrease of sharp winkles of graphene film after the secondary transfer. What is more, we can see that the secondary transfer process almost did not reduce the performance of FOLED by comparing the current efficiency of D1 and D2; a series of repetitive experiments also support this conclusion. We further improved the brightness and efficiency of FOLED by introducing the modified layer PEDOT:PSS as the D3 showed. The luminance of the D3 can reach 35000 cd/m^2^, and the maximum current efficiency was 16.19 cd/A, which was higher than the D2 of 10.74 cd/A. That is because PEDOT:PSS played a role as work function stair and enhanced the sheet conductivities. Furthermore, it also smoothed the surface of secondary-transferring SLG film by filling part of the “Valley,” making the FOLED more stable.

## Conclusion

In this paper, we explored in detail the sharp wrinkles of graphene duplicating the grain boundary cracks of copper foil after the first bubble transfer; the sharp wrinkles can cause a large surface roughness, resulting in deterioration even breakdown of FOLED. We proposed a secondary-transferring method to re-transform the wrinkles on graphene surface into “Valley” form to fabricate the stable FOLED; the graphene film is almost nondestructively transferred by controlling the different adhesiveness. The maximum luminance can reach about 35000 cd/m^2^, and maximum current efficiency was 16.19 cd/A with PEDOT:PSS/SLG/NOA63 framework. This method can also be applied to prepare large area high-quality graphene by roll-to-roll way.

## Additional file

Additional file 1: **Figure S1.** The count of Ig’/Ig as shown in the following figure. Most of the Ig’/Ig values are concentrated at 1.75 and the standard deviation was 0.015, which shows the consistency of the sample quality, while somewhere the Ig’/Ig was ~ 0.8, which illustrates bilayer of graphene sample and the monolayer rate of our graphene sample is > 90%. (DOCX 106 kb)

## Abbreviations

CVD: Chemical vapor deposition
FOLED: Flexible organic light-emitting device
HOMO: Highest occupied molecular orbital
HRA: Heat release adhesive
ITO: Indium tin oxide
LUMO: Lowest unoccupied molecular orbital
SLG: Single-layer graphene
