# Supplementary material for: Secondary-Transferring Graphene Electrode for Stable FOLED
Source: Nanoscale Res Lett. 2018 Nov 6;13:352. doi: 10.1186/s11671-018-2767-z (PMC6219997; doi:10.1186/s11671-018-2767-z)
Supplement: Supplementary file 1 — Figure S1. The count of Ig’/Ig as shown in the following figure. Most of the Ig’/Ig values are concentrated at 1.75 and the standard deviation was 0.015, which shows the consistency of the sample quality, while somewhere the Ig’/Ig was ~ 0.8, which illustrates bilayer of graphene sample and the monolayer rate of our graphene sample is > 90%. (DOCX 106 kb) [file 11671_2018_2767_MOESM1_ESM.docx]

Yunjie Teng^1^, Shoufeng Tong^2^, and Min Zhang ^2^*

^1^College of Opto-Electronic Engineering, Changchun University of Science and Technology, Changchun, 130012, People’s Republic of China.

^2^Institute of Space Photo-Electronic Technology, Changchun University of Science and Technology, Changchun, 130012, People’s Republic of China.


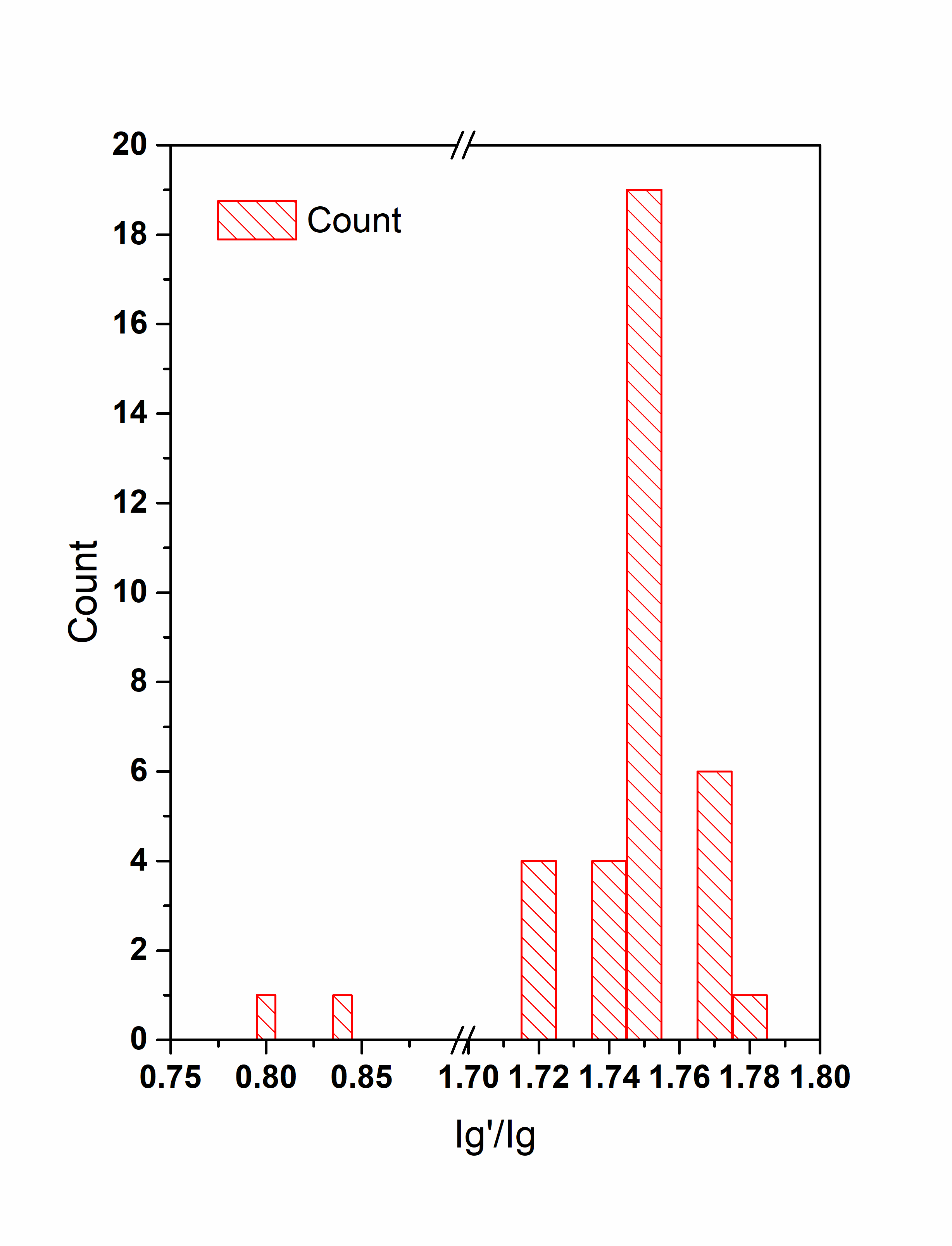


Figure S1. The count of Ig'/Ig as shown in the following figure. Most of the Ig'/Ig values are concentrated at 1.75 and the standard deviation was 0.015, which shows the consistency of the sample quality, while somewhere the Ig'/Ig was ~0.8, which illustrates bilayer of graphene sample and the monolayer rate of our graphene sample is >90%.
